# Good mid‐term outcomes and over 80% survivorship rate after all‐inside repair of bucket‐handle and full‐thickness radial tears

**DOI:** 10.1002/jeo2.70721

**Published:** 2026-04-16

**Authors:** Lika Dzidzishvili, Luca Ambrosini, Federico Maria Adravanti, Claudio Rossi, Maria Pia Neri, Ashraf Hantouly, Stefano Zaffagnini, Alberto Grassi

**Affiliations:** ^1^ Department of Orthopaedic Surgery and Traumatology Universidad Autónoma de Barcelona, Hospital Universitari Germans Trias i Pujol Barcelona Spain; ^2^ Clinica Ortopedica e Traumatologica II IRCCS Istituto Ortopedico Rizzoli Bologna Italy; ^3^ Orthopedic Surgery Department McMaster University Hamilton Ontario Canada; ^4^ Dipartimento di Scienze Biomediche e Neuromotorie (DIBINEM) University of Bologna Bologna Italy

**Keywords:** ACL reconstruction, all‐inside repair, bucket handle meniscus tear, meniscus repair, radial meniscus tear

## Abstract

**Purpose:**

To evaluate failure rates, survivorship, patient‐reported outcome measurements (PROMs), and prognostic factors following all‐inside arthroscopic repair of bucket‐handle tears (BHT) and full‐thickness radial meniscal tears (RT).

**Methods:**

A retrospective cohort study included consecutive patients undergoing all‐inside repair of BHT or RT by a single surgeon (2019–2025) with a minimum 2‐year follow‐up. Inclusion required primary repair using all‐inside devices. Demographic data, tear morphology, chronicity, and concomitant procedures were recorded. Failure was defined as surgical re‐intervention or magnetic resonance imaging‐confirmed re‐tear. PROMs included subjective International Knee Documentation Committee (IKDC), Tegner Activity Scale, Patient Acceptable Symptom State (PASS), and Visual Analog Scale (VAS) scores. Return to sport (RTS) rates and the achievement of IKDC‐PASS threshold were assessed. Kaplan–Meier survival analyses for each lesion type were performed at 1, 2, 3, and 4 years; subgroup comparisons (location, ACL reconstruction, aetiology) were performed using the log‐rank test.

**Results:**

Fifty‐five repairs (34 BHTs, 21 RTs; 72.7% males; mean age 27.0 ± 11.7 years) were analysed. BHTs were medial in 24 cases and lateral in 10; all RTs were lateral. Overall, 52.7% were chronic, 54.6% treated with concomitant ACL reconstruction, and 83.7% were traumatic. Five repairs failed (mean 22.3 ± 11.9 months), yielding cumulative failure rates of 0.0% at 1 year, 6.0% at 2 years, 8.7% at 3 years and 17.8% at 4 years. Failures were more frequent in medial BHTs (35.5%), degenerative tears (*p* = 0.0194) and isolated repairs (*p* = 0.0321). Overall satisfaction was 97.9%, with 87.2% patients returning to sport and 48.9% reaching their preinjury level.

**Conclusions:**

Meniscal repair of BHTs and RTs achieved 98% patient satisfaction and high return‐to‐sport rates with a 17% mid‐term failure. Medial BHTs and degenerative lesions demonstrated a higher risk of failure, whereas repairs performed concomitantly with ACL reconstruction showed lower failure rates. These findings support early repair and highlight the importance of tear pattern, aetiology, lesion chronicity and concomitant procedures in predicting outcomes.

**Level of Evidence:**

Level IV, therapeutic case series.

**Abbreviations**
ACL: anterior cruciate ligament
AI: all‐inside technique
BHT: bucket handle tear
CI: confidence interval
HR: hazard ratio
IKDC: International Knee Documentation Committee
IO: inside‐out technique
IQR: interquartile range
LMORT: lateral meniscal oblique radial tear
MRI: magnetic resonance imaging
PASS: Patient Acceptable Symptom State
PROMs: patient‐reported outcome measurements
RT: radial tear
RTS: return to sport
VAS: Visual Analog Scale

## INTRODUCTION

The menisci are crescent‐shaped fibrocartilaginous structures of the knee that play a critical role in load distribution, joint stability and congruity [8, 13]. While meniscal tears are common, complex injuries such as bucket‐handle tears (BHTs) and radial tears (RTs) are particularly concerning [4, 8, 9]. BHTs, characterised by a longitudinal split with displacement of a central fragment, can severely disrupt knee biomechanics, leading to pain, locking, instability and reduced range of motion [4, 6]. RTs involve disruption of the circumferential collagen fibres that generate hoop stresses, converting axial loads into tensile strain [7, 8]. Untreated, both BHTs and RTs significantly compromise joint biomechanics, emphasising the importance of early detection and timely repair to restore near‐native knee loading and potentially slow osteoarthritis progression [8, 9, 30].

Repair of BHTs has been associated with higher failure rates compared with other meniscal tear types, likely due to multifactorial challenges, while RTs have historically been considered irreparable and managed with partial meniscectomy, particularly when occurring in the avascular inner meniscus [4, 8, 9, 23]. Over the past decades, surgical management of complex meniscal tears has evolved to preserve meniscal anatomy while improving outcomes [8, 9]. Traditionally, inside‐out (IO) repair was the gold standard; however, it is more invasive and carries a small risk of neurovascular injury [23]. The all‐inside (AI) technique has emerged as a minimally invasive alternative, reducing surgical trauma and complication rates [6, 23]. Despite these advantages, evidence regarding outcomes of AI repair for complex meniscal tears remains limited.

Therefore, the purpose of the present study was to evaluate failure rates and patient‐reported outcome measurements (PROMs) following AI repair of BHTs and full‐thickness RTs. The authors hypothesised that outcomes would be comparable between BHT and RT repairs.

## METHODS

### Study design and patients selection

This retrospective comparative study was conducted at IRCCS Istituto Ortopedico Rizzoli, Bologna, Italy, and included patients treated between January 2019 and December 2025, all of whom were operated on by a single surgeon, A.G.Eligible participants were adults (≥ 18 years) who sustained traumatic or degenerative RT and BHT meniscal tears deemed suitable for repair. Patients older than 60 years, those with multiligamentous injuries, or those with advanced preexisting degenerative changes (Kellgren‐Lawrence grade III–IV) were excluded. The study was approved by the local Ethics Committee (approval number 645/2025/Oss/IOR). All surgical procedures were performed according to standard clinical practice, and the study adhered to the ethical principles outlined in the Declaration of Helsinki. This study was conducted and reported in accordance with the Strengthening the Reporting of Observational Studies in Epidemiology (STROBE) guidelines (Supporting Information: File S1) [28].

All clinically and magnetic resonance imaging (MRI)‐confirmed Radial and bucket‐handle meniscal tears, regardless of patient age, time from injury to surgery, degenerative versus traumatic nature of the tear, or concomitant injuries (e.g., ACL tear), were considered for meniscal repair. All patients were thoroughly informed preoperatively about the possible prognosis, failure rates of specific tear types, and differences in rehabilitation protocols and return‐to‐sport timing between repair and meniscectomy. The following rationale was then applied for inclusion in the present study: repair was attempted in all lesions except for nonrepairable white‐white zone BH or RTs, irreducible lesions, BH tears with radial cleavage of the body, or cases in which the patient declined repair.

Only patients who had meniscal repair with at least 2 years of follow‐up were assessed. Patients with shorter follow‐up, who were included in the screening process to avoid selection bias, or who underwent meniscectomy, were excluded.

### Meniscal lesions' arthroscopic assessment and treatment

**Figure 1:**
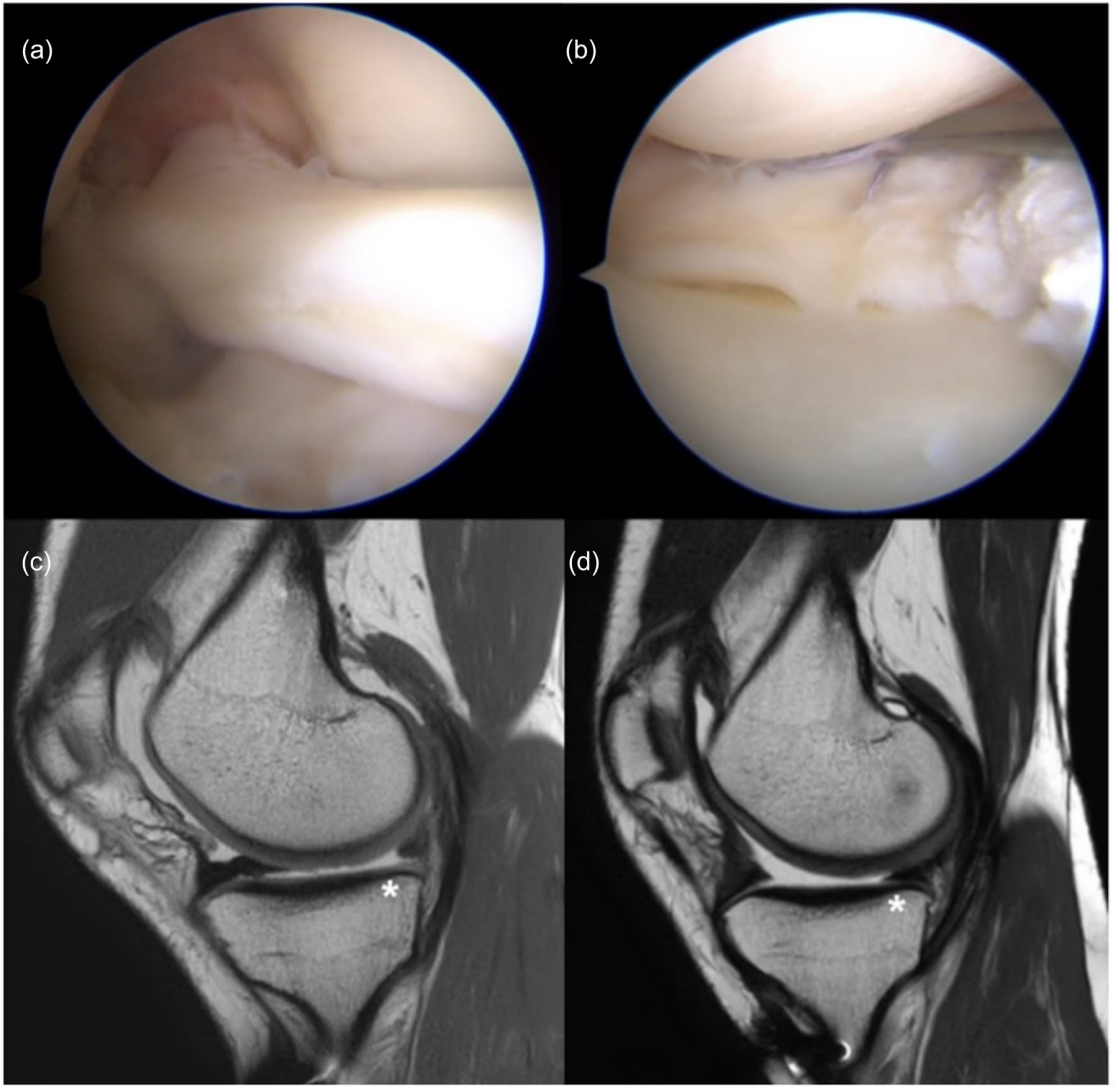
Medial bucket handle repair. Arthroscopic and MRI images of a medial bucket‐handle meniscal tear (a, c), repaired using the all‐inside technique (b). Postoperative follow‐up MRI demonstrates complete healing (d). MRI, magnetic resonance imaging.

The types of meniscal injuries were assessed by a single surgeon after a systematic and standardised arthroscopic assessment. Bucket handle tears, either medial (Figure 1) or lateral, were considered in the case of a dislocated meniscus within the notch. These lesions were treated by debridement of the lesion bed and repair with vertical stitches using AI devices (Truespan DePuy‐Mitek or Ultra‐FasTfix Smith & Nephew). Meniscoplasty or release from adhesions within the notch was performed if the lesion was not reducible only by probing. Patients requiring additional outside‐in stitches were excluded from the final analysis.

**Figure 2:**
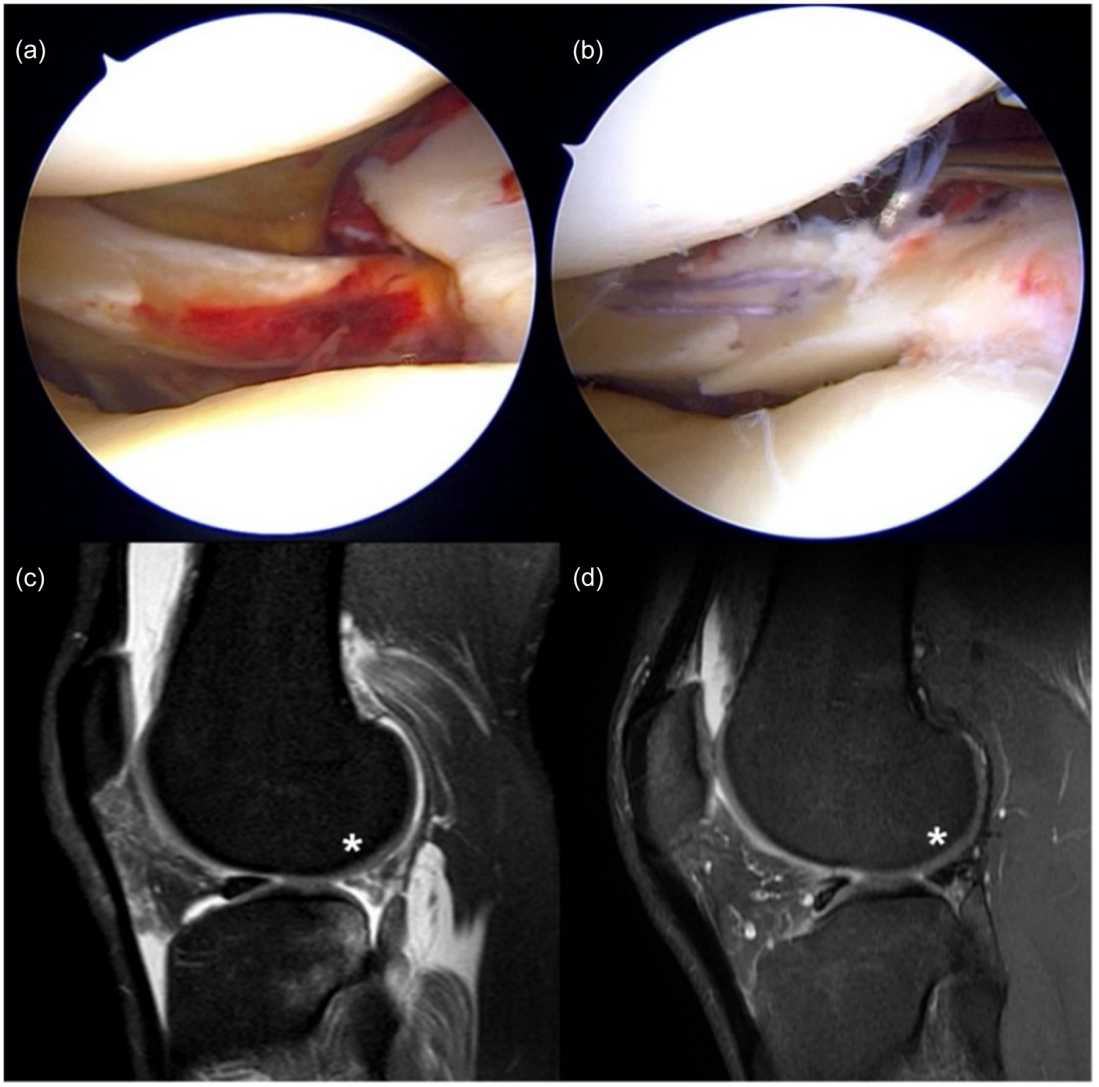
Radial tear of the lateral meniscus posterior horn repair. Arthroscopic and MRI findings demonstrating a lateral meniscus oblique radial (LMOR) tear (a, c), repaired using the all‐inside technique (b). Postoperative Follow‐up MRI demonstrates complete healing (d). MRI, magnetic resonance imaging.

**Figure 3:**
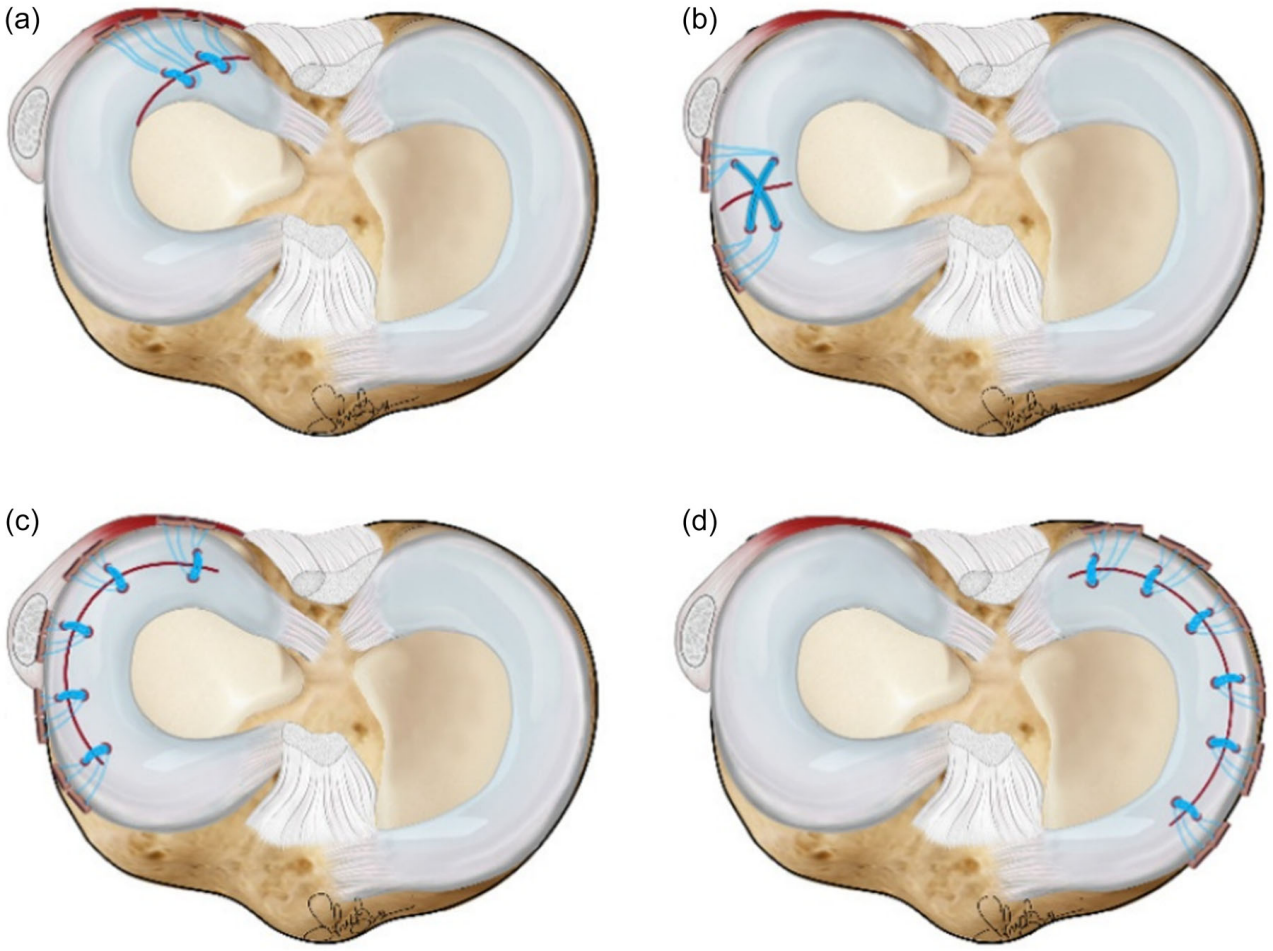
Schematic representation of meniscal suture configuration for Radial tear of the posterior horn (a) and body (b), and form lateral (c) and medial (d) Bucket handle tears.

RTs were considered in the case of a complete lesion of the meniscal structure extending from the free meniscal margin to the joint capsule. These lesions were classified based on the location: meniscal body or posterior horn (Figure 2). The radial lesion involving the posterior horn usually had an oblique‐radial course and was also defined as lateral meniscal oblique radial tears (LMORT) type III and IV [10]. These lesions were repaired with 1 or 2 horizontal stitches using AI devices (Truespan DePuy‐Mitek or Ultra‐FasTfix Smith & Nephew) (Figure 3).

Medial and lateral root lesions, according to the LaPrade classification [11], were not included in the present series.

### Patients' assessment

The laterality and type of tear, the nature of the injury or type of trauma, the timing from injury to surgery and other concomitant procedures were obtained from medical records. Specifically, a degenerative tear was defined in the case of the absence of a clear trauma, while a chronic tear was defined when surgical treatment was delayed for more than 3 months since diagnosis or symptoms onset. Based on these features, patients were dichotomised based on types of tear (bucket handle vs. radial), laterality of lesion (medial vs. lateral), concomitant ACL reconstruction, aetiology of the tear (traumatic vs. degenerative) and chronicity of the lesion (<3 vs. >3 months) [9, 30].

The hospital database was searched for reoperations. Moreover, an online survey was sent to all the patients included in order to investigate further traumas or surgeries. The Tegner Activity Scale and the subjective International Knee Documentation Committee (IKDC) score were collected as well. The number of patients achieving the threshold PASS value of the IKDC score according to Maheshwer et al. (69.0 points) was calculated [12]. Pain and overall knee function were graded from 0 to 10, while satisfaction and returning to sport were assessed in a dichotomic manner (Yes or No).

### Statistical analysis

Statistical analysis was performed with MedCalc (MedCalc Software, Acacialaan, 22 Ostend, Belgium). Normal distribution of continuous variables was assessed with the Shapiro–Wilk test. Continuous variables with normal distribution were reported as mean ± standard deviation, while median with interquartile range was used in the case of nonnormal distribution. Categorical variables were reported as absolute number and proportion of the total sample with 95% confidence intervals (CI). An independent sample *t*‐test was used to compare the continuous variables with normal distribution, while the Mann–Whitney *U*‐test was used in the case of nonnormal distribution. The Fisher exact test and the Chi‐squared test were used to compare dichotomous categorical variables.

Kaplan–Meier curves were created for each lesion type using the failure (defined as reoperation or re‐injury at MRI) as the primary endpoint. The cumulative failure rate was calculated at 1‐, 2‐, 3‐ and 4‐year intervals. The log‐rank test was used to compare the failure rate of different patients' subgroups. Statistical significance was set with *p* < 0.05.

## RESULTS

**Figure 4:**
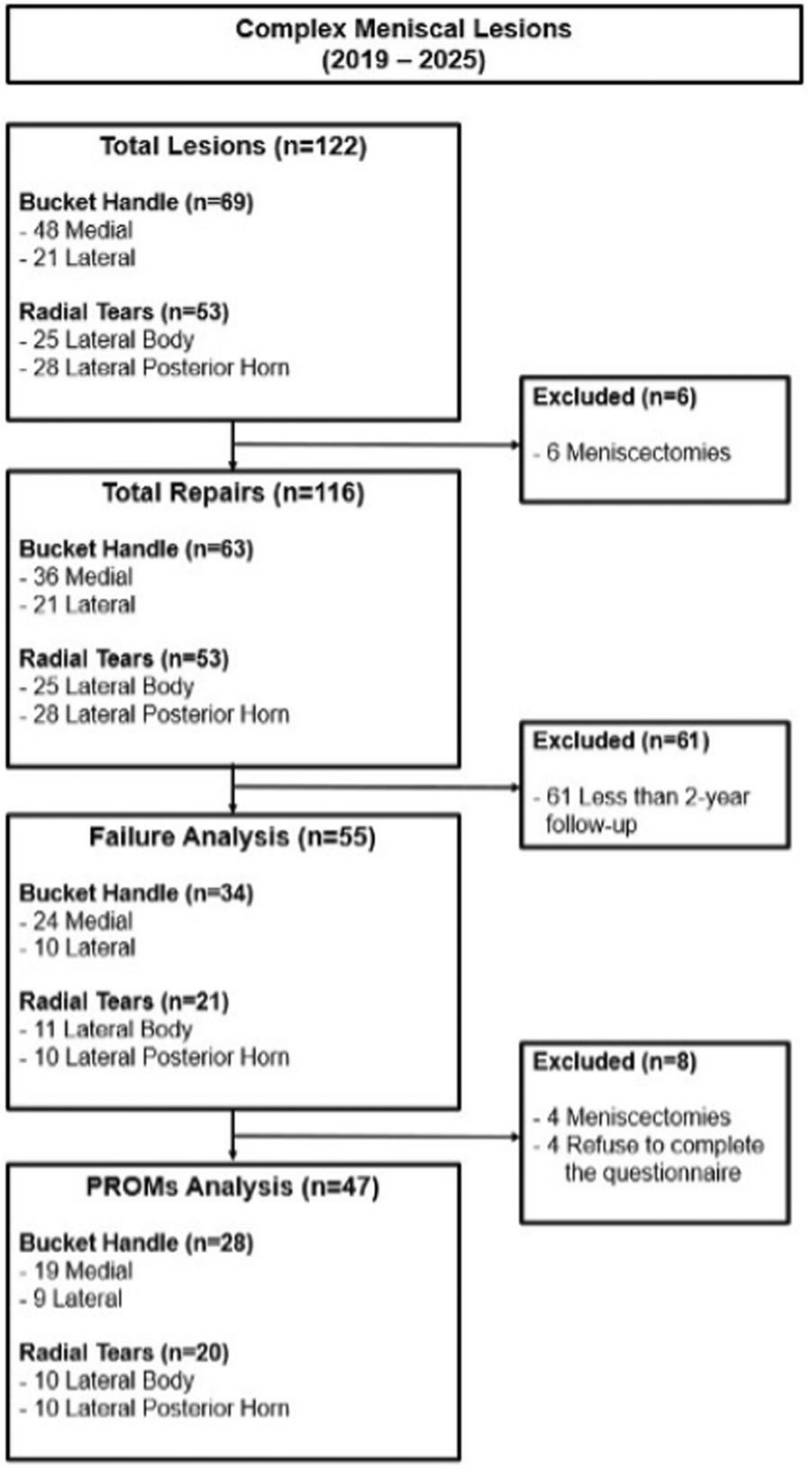
Flow‐chart of patient inclusion. PROMs, patient‐reported outcome measurements.

Between 2019 and 2025, 122 meniscal BHT and RT lesions were identified. After excluding six cases (5%) treated with meniscectomy, 116 lesions underwent meniscal repair and were included in the study. Of these, 63 lesions (54.3%) were BHT and 53 lesions (45.7%) were full‐thickness RTs of the lateral meniscus. After excluding patients with fewer than 2 years of follow‐up and those with additional outside‐in stitches, 55 repaired lesions were available for failure analysis: 34 BHT (61.8%) and 21 RTs (38.2%). Patient selection and exclusion criteria are summarised in Figure 4.

BHT involved the medial meniscus in 24 cases (43.6%) and the lateral meniscus in 10 cases (18.2%). RTs were exclusively lateral, involving the body in 11 cases (20.0%) and the posterior horn in 10 cases (18.2%) (Table 1).

**Table 1:** Patients characteristics according to type of lesion.

Total patients	Total *n*°	Age (years)	Sex (male/female)	Concomitant ACL‐R	Traumatic	Chronic	Time to surgery (months)
	55	27.0 ± 11.7	40 (72.7%)/15 (27.3%)	30 (54.6%)	46 (83.7%)	29 (52.7%)	5.7 ± 9.4
Type of tear							
Bucket handle medial	24	24.9 ± 10.3	13 (54.2%)/7 (45.8%)	11 (45.8%)	17 (70.8%)	11 (45.8%)	4.5 ± 6.3
Bucket handle lateral	10	27.8 ± 14.3	8 (80.0%)/2 (20.0%)	4 (40.0%)	8 (80.0%)	3 (30.0%)	2.7 ± 2.9
Radial lateral meniscus body	11	29.1 ± 10.5	10 (90.9%)/1 (8.1%)	5 (45.5%)	11 (100%)	8 (72.7%)	5.3 ± 3.5
Radial‐oblique lateral meniscus PH	10	28.7 ± 12.8	9 (90.0%)/1 (10.0%)	10 (100%)	10 (100%)	7 (70.0%)	5.8 ± 5.5
Radial versus bucket handle							
Bucket handle	34	25.7 ± 11.5	22 (64.7%)/12 (35.3%)	15 (44.1%)	25 (73.5%)	14 (41.2%)	3.9 ± 5.5
Radial	21	28.4 ± 11.3	19 (90.5%)/2 (9.5%)	15 (71.4%)	21 (100.%)	15 (71.4%)	5.5 ± 4.5
Meniscus laterality							
Medial	24	24.9 ± 10.3	13 (54.2%)/7 (45.8%)	11 (45.8%)	17 (70.8%)	11 (45.8%)	4.5 ± 6.3
Lateral	31	28.2 ± 12.1	27 (87.1%)/4 (12.9%)	19 (61.3%)	29 (93.6%)	18 (58.1%)	4.6 ± 4.2
Concomitant ACL‐R							
Yes	30	28.1 ± 12.0	21 (70.0%)/9 (30.0%)	‐	31 (100%)	19 (63.3%)	5.6 ± 6.2
No	25	25.1 ± 10.6	19 (76.0%)/6 (24.0%)	‐	16 (64.0%)	10 (40.0%)	3.1 ± 2.4
Traumatic							
Yes	46	26.1 ± 11.1	36 (78.2%)/10 (21.8%)	30 (65.2%)	‐	26 (56.5%)	4.8 ± 5.3
No	9	27.1 ± 11.2	4 (44.4%)/5 (55.6%)	0 (0.0%)	‐	3 (33.3%)	2.9 ± 2.8
Chronic (>3 months)							
Yes	29	27.0 ± 10.8	23 (79.3%)/6 (20.7%)	19 (65.5%)	26 (89.7%)	‐	7.7 ± 5.7
No	26	26.5 ± 12.3	17 (65.4%)/9 (34.6%)	11 (42.3%)	20 (76.9%)	‐	1.4 ± 0.6

*Note*: Table 1. Patients' characteristics and demographics of the whole series of the different subgroups.

Abbreviations: ACL‐R, anterior cruciate ligament reconstruction; PH, posterior horn.

The cohort was predominantly male (72.7%), with females comprising 27.3%, and had a mean age at surgery of 27.0 ± 11.7 years. Nearly half of the lesions were associated with concomitant ACL reconstruction (54.6%) and were chronic (52.7%), with a mean time from injury to surgery of 5.7 ± 9.4 months. The majority of cases (83.7%) were of traumatic aetiology (Table 1).

### Failure rate and survivorship

**Figure 5:**
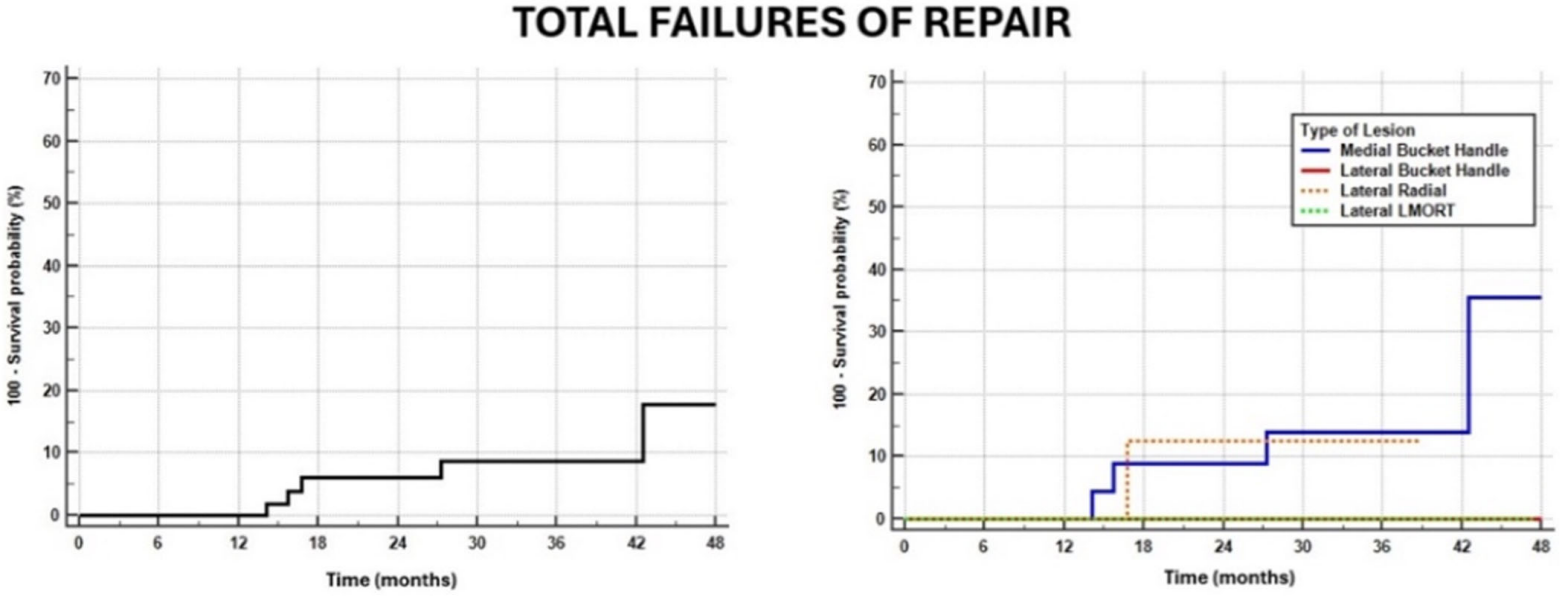
Failure rate of the whole series and of the different lesion subtypes.

All 55 lesions were evaluated at an average follow‐up of 37.2 ± 14.6 months. A total of five lesions (four medial bucket handle and one RT of the lateral meniscus body) were considered failed after an average of 22.3 ± 11.9 months; the four medial tears underwent partial meniscectomy, while the radial lateral tear was found re‐torn at MRI, and the patient decided not to undergo reoperation despite symptoms. One patient had septic arthritis 3 months after the repair of a chronic bucket handle tear, due to contamination from the skin incision. This patient underwent joint lavage, but it was not considered a failure of the repair. Thus, the overall failure rate was 0.0% at 1‐year, 6.0% at 2‐year, 8.7% at 3‐year, and 17.8% at 4‐year (Table 2). Despite not being significantly different, a higher failure rate was reported for medial bucket handle tears (16.7%), with respect to posterior horn RTs (0.0%) and RTs of the meniscal body (9.1%) (Figure 5).

**Table 2:** Survivorship of meniscal repair according to variables.

	Survival time (years)					
	Patient *n*°	1‐year	2‐year	3‐year	4‐year	Statistics
Total failures	5/55 (9.1%)	0.0% (SE 0.0%)	6.0% (SE 1.2%)	8.7% (SE 4.2%)	17.8% (SE 9.5%)	
Type of tear						*p* = 0.3076
Bucket handle medial	4/24 (16.7%)	0.0% (SE 0.0%)	8.9% (SE 6.0%)	14.0% (SE 7.5%)	35.5% (19.5%)	Ref.
Bucket handle lateral	0/10 (0.0%)	0.0% (SE 0.0%)	0.0% (SE 0.0%)	0.0% (SE 0.0%)	0.0% (SE 0.0%)	HR = NA
Radial lateral meniscus body	1/11 (9.1%)	0.0% (SE 0.0%)	12.5% (11.7%)	12.5% (11.7%)	12.5% (11.7%)	HR = 0.80 (0.60–11.50)
Radial‐oblique lateral meniscus PH	0/10 (0.0%)	0.0% (SE 0.0%)	0.0% (SE 0.0%)	0.0% (SE 0.0%)	0.0% (SE 0.0%)	HR = NA
Tear type						*p* = 0.6171
Bucket handle	4/34 (11.8%)	0.0% (SE 0.0%)	6.4% (SE 4.3%)	10.0% (SE 5.6%)	20.0% (SE 10.6%)	HR = 1.7 (0.25–11.58)
Radial	1/21 (4.8%)	0.0% (SE 0.0%)	6.2% (SE 6.0%)	6.2% (SE 6.0%)	6.2% (SE 6.0%)	HR = 0.58 (0.08–3.96)
Meniscus laterality						*p* = 0.1084
Medial	4/24 (16.7%)	0.0% (SE 0.0%)	8.9% (SE 6.0%)	14.0% (SE 7.5%)	35.5% (SE 19.5%)	HR = 0.20 (0.03–1.16)
Lateral	1/31 (3.2%)	0.0% (SE 0.0%)	4.0% (SE 3.9%)	4.0% (SE 3.9%)	4.0% (SE 3.9%)	HR = 5.00 (0.86–29.20)
Concomitant ACL‐R						*p* = 0.0321*
Yes	0/30 (0.0%)	0.0% (SE 0.0%)	0.0% (SE 0.0%)	0.0% (SE 0.0%)	0.0% (SE 0.0%)	HR = NA
No	5/25 (20.0%)	0.0% (SE 0.0%)	12.3% (SE 3.4%)	17.2% (SE 4.2%)	29.0% (SE 1.3%)	HR = NA
Traumatic						*p* = 0.0194*
Yes	2/46 (4.3%)	0.0% (SE 0.0%)	4.8% (SE 3.3%)	4.8% (SE 3.3%)	4.8% (SE 3.3%)	HR = 0.15 (0.02–1.46)
No	3/9 (33.3%)	0.0% (SE 0.0%)	12.5% (SE 11.7%)	27.3% (SE 16.5%)	51.4% (SE 22.7%)	HR = 6.31 (0.68–58.43)
Chronic (>3 months)						*p* = 0.8378
Yes	2/29 (6.9%)	0.0% (SE 0.0%)	8.1% (SE 5.5%)	8.1% (SE 5.5%)	8.1% (SE 5.5%)	HR = 0.83 (0.14–4.85)
No	3/26 (11.5%)	0.0% (SE 0.0%)	3.8% (SE 3.7%)	8.9% (SE 6.1%)	21.9% (SE 13.1%)	HR = 1.20 (0.20–7.01)

*Note*: Table 2. Failure rate of meniscal repair of the whole series and of different subgroups.

Abbreviations: ACL‐R, anterior cruciate ligament reconstruction; HR, hazard ratio; NA, not assessed; PH, posterior horn; SE, standard error.

* *p* < 0.05.

**Figure 6:**
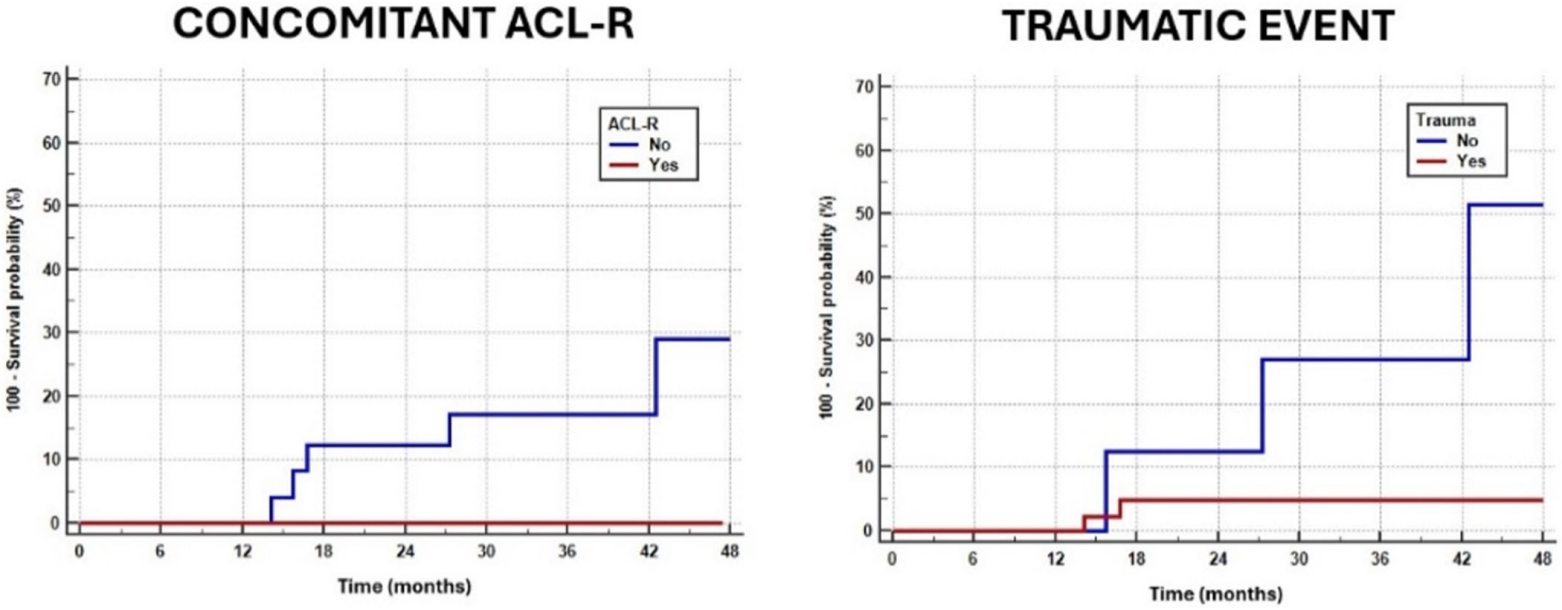
Comparison of failure rate of the subgroups based on concomitant ACL reconstruction (left) and based on traumatic versus degenerative aetiology of the tear (right). Both graphs show significant differences between the red and blue curves (*p* < 0.05). ACL, anterior cruciate ligament.

According to the survivorship analysis of the different subgroups, a higher hazard ratio for failure (HR = 6.31) was reported for patients with degenerative tears respect to those with traumatic tears (51.4% vs. 4.8%; *p* = 0.0194) and for those with isolate repair respect to those with concomitant ACL reconstruction (29.0% vs. 0.0%; *p* = 0.0321) (Table 2) (Figure 6). No significant differences were reported for tear type, meniscus laterality and timing of repair.

### PROMs

The PROMs were not assessed in eight patients who underwent meniscectomy due to surgical failure prior to getting a minimum of 2 years of follow‐up, or who refused to complete the questionnaires; therefore, the PROMs analysis was performed in 47 patients (85.5%).

A total of 97.9% patients were satisfied with the surgical treatment, 87.2% returned to sport after the procedure, and 48.9% returned to the preinjury level of activity (Table 3). Patients returned to sport after an average of 8.5 ± 5.3 months, with shorter time in those with isolated repair compared to those with concomitant ACL reconstruction (5.5 ± 3.7 months vs. 11.0 ± 5.7 months; *p* = 0.0012).

**Table 3:** Subjective evaluation according to lesion type and patients characteristics.

Total patients	Completed PROMs	Satisfaction (Yes)	Return to sport (Yes)	Pain (0–10)	Function (0–10)	Tegner	Subjective IKDC	PASS IKDC
	47/55 (85.5%)	46 (97.9%)	41 (87.2%)	2.3 ± 2.1	7.5 ± 2.0	5 (2–8)	76.5 ± 15.2	35 (74.5%)
Type of tear								
Bucket handle medial	19/24 (79.2%)	19 (100%)	16 (84.2%)	1.9 ± 1.9	7.7 ± 2.0	3 (2–6)	78.2 ± 12.8	15 (78.9%)
Bucket handle lateral	9/10 (90.0%)	9 (100%)	8 (88.9%)	1.6 ± 1.8	7.7 ± 2.1	5 (2–6)	78.7 ± 17.6	7 (77.8%)
Radial lateral meniscus body	10/11 (90.9%)	9 (90.0%)	9 (90.0%)	3.2 ± 2.3	6.6 ± 2.5	7 (5–9)	70.1 ± 18.1	5 (50.0%)
Radial‐oblique lateral meniscus PH	10/10 (100%)	10 (100%)	9 (90.0%)	2.8 ± 2.3	7.7 ± 1.4	5 (3–8)	77.9 ± 14.5	8 (80.0%)
Radial versus bucket handle								
Bucket handle	28/34 (82.4%)	28 (100%)	24 (85.7%)	1.8 ± 1.8	7.7 ± 2.0	3 (2– 6)	78.4 ± 14.2	22 (78.6%)
Radial	20/21 (95.2%)	19 (95.0%)	17 (89.5%)	3.0 ± 2.2	7.2 ± 2.0	6 (4–9)	74.0 ± 16.5	13 (65.0%)
Meniscus laterality								
Medial	19/24 (79.2%)	19 (100%)	16 (84.2%)	1.9 ± 1.9	7.7 ± 2.0	3 (2–6)	78.2 ± 12.8	15 (78.9%)
Lateral	29/31 (93.6%)	28 (96.6%)	26 (89.7%)	2.6 ± 2.2	7.3 ± 2.1	6 (2–8)	75.5 ± 17.6	20 (69.0%)
Concomitant ACL‐R								
Yes	28/30 (93.3%)	28 (100%)	24 (85.7%)	2.7 ± 2.2	7.5 ± 1.8	5 (2–8)	75.2 ± 17.2	19 (67.9%)
No	20/25 (80.0%)	19 (95.0%)	19 (95.0%)	2.5 ± 2.1	7.4 ± 2.1	6 (2–6)	75.4 ± 15.6	16 (80.0%)
Traumatic								
Yes	40/44 (90.9%)	39 (97.5%)	35 (87.5%)	1.6 ± 2.5	7.5 ± 2.1	5 (2–8)	76.5 ± 16.0	29 (72.5%)
No	7/9 (77.8%)	7 (100%)	6 (85.7%)	2.5 ± 2.2	7.4 ± 1.3	2 (2–5)	76.5 ± 9.9	6 (85.7%)
Chronic (>3 months)								
Yes	24 (82.8%)	23 (95.8%)	21 (87.5%)	3.0 ± 1.8	7.0 ± 1.8	5 (2–8)	72.6 ± 14.5	13 (54.1%)
No	24 (96.0%)	24 (100%)	22 (87.5%)	1.6 ± 2.1	8.0 ± 2.1	6 (2–7)	80.7 ± 15.1	22 (91.7%)

*Note*: Table 3. Subjective assessment and PROMs of the whole series and of different subgroups.

Abbreviations: ACL‐R, anterior cruciate ligament reconstruction; IKDC, International Knee Documentation Committee; PASS, Patient Acceptable Symptom State; PH, posterior horn; PROMs, patient‐reported outcome measurements.

The postoperative median Tegner Activity Scale was 5 (IQR: 2–8), which was significantly lower compared to the median preinjury value of 7 (IQR: 4–9) (*p* = 0.0212). Patients with BHT and with medial‐sided lesions had a lower postoperative Tegner score compared to those with RTs (3, IQR: 2–6 vs. 6, IQR: 4–9; *p* = 0.0410) and with lateral lesions (3, IQR: 2–6 vs. 6, IQR: 2–8; *p* = 0.0443) (Table 3).

The average IKDC at final follow‐up was 76.5 ± 15.2 points, ranging from 70.1 ± 18.1 to 80.7 ± 15.1 according to the various lesion subtypes or different patients' subgroups, without any significant differences (*p* > 0.05) (Table 3). However, a significantly lower number of patients with chronic tears achieved the IKDC PASS with respect to those treated in the acute setting (54.1% vs. 91.7%; *p* = 0.0094).

The overall knee function was rated on average as 7.5 ± 2.0 points without significant differences among subgroups, while knee pain was rated on average as 2.3 ± 2.1 points. Patients with RTs and with chronic lesions had higher pain compared to those with bucket handle tears (3.0 ± 2.2 vs. 1.8 ± 1.8; *p* = 0.0436) and with acute lesions (3.0 ± 1.8 vs. 1.6 ± 2.1; *p* = 0.0169) (Table 3).

## DISCUSSION

The main finding of this study is that repair using AI devices for complex meniscal tears, including BHT and full‐thickness RTs, achieved over 80% survivorship, with significant improvements in PROMs at short‐ to mid‐term follow‐up, as well as a high rate of return to sport (RTS). However, the study hypothesis was only partially confirmed, as outcomes varied according to tear type, concomitant ACL reconstruction, and traumatic versus degenerative aetiology. Notably, medial‐sided BHT and degenerative lesions demonstrated higher failure rates, particularly when repaired in isolation, whereas repairs performed concomitantly with ACL reconstruction were associated with lower failure rates.

Failure rates following repairs of complex meniscal tears, such as BHTs and RTs, have been a topic of active debate over recent decades [4, 6]. Although the ‘save the meniscus’ movement has been widely adopted and supported by current evidence [21], reported failure rates in the literature vary considerably, and the available data remain a concern for many knee surgeons.

Another important aspect of this debate is the choice of repair technique [6]. Despite variability in definitions and reporting of failure across studies, most data suggest that overall failure rates are relatively high and comparable among different repair techniques [4, 6]. Evaluating outcomes using a single repair technique may therefore help clarify existing controversies.

In the present study, all repairs were performed using the AI technique. The overall failure rates were 0% at 1 year, 6% at 2 years, 8.7% at 3 years and 17.8% at 4 years. The absence of early failures may seem unexpected; however, previous studies have reported that most failures occur within the first 2 years after BHT repair [4]. Similarly, Saltzman et al. reported that failure rates tend to increase up to approximately 15 months postoperatively [22]. This evidence supports our rationale for setting a minimum follow‐up period of 2 years to better capture the true rate of postoperative failure.

A meta‐analysis previously demonstrated a significantly higher failure rate following BHT repair compared with other meniscal tear types, reporting a pooled failure rate of 14.8% [4]. In that analysis, 30.8% of failures occurred within 6 months of repair, 61.5% between 6 and 24 months, and 7.7% beyond 24 months. It should be noted, however, that various repair techniques were included across the included studies [4]. Additionally, a retrospective case series using the IO technique reported failure rates of 6.4% at 6 months, 15.4% at 1 year, 21.6% at 2 years and 30.1% at 3 years following BHT repair [22].

It is noteworthy that only one failed lateral RT was observed among patients in the midbody lateral meniscus RT group. Over the past decades, the AI repair technique has gained considerable popularity due to its advantages, including shorter operative time, reduced risk of iatrogenic neurovascular injury, and technical ease. The biomechanical superiority of the AI technique for RTs has been well described in the literature; however, clinical validation has been limited [2, 18]. The findings of the present study corroborate these biomechanical results, suggesting that higher survivorship and lower failure rates can be expected following lateral meniscus RT repair using the AI technique.

In the present study, degenerative and medial BHTs demonstrated higher failure rates, with four of the five failures (80%) occurring in medial BHT repairs. Despite this, the overall failure rate observed in our cohort (16.7%) was lower than those reported in previous studies [1, 4, 6]. It should be acknowledged that, in the present study, the indications for meniscal repair were strict and proactive, particularly for medial tears. In fact, 95% of all BHT and full‐thickness RTs were treated with repair.

This observation is consistent with previous biomechanical studies showing that the medial meniscus experiences greater loading and contact stresses compared with the lateral meniscus [27]. Furthermore, the increased anatomical mobility of the lateral meniscus enhances its ability to accommodate altered tibiofemoral biomechanics, thereby reducing the likelihood of repair failure. In addition, involvement of the lateral meniscus was associated with superior functional outcomes at the final follow‐up [17]. Consistent with this finding, Tucciarone et al. observed that all failures (7.5%) in their cohort of 40 patients involved the medial meniscus, all of which subsequently required partial meniscectomy [26].

Although the present study is retrospective in nature, our findings suggest that, despite medial‐sided and degenerative BHT repairs being traditionally considered poor prognostic factors, postoperative failure following AI repair may occur at lower rates, particularly during the early postoperative period. In such cases, in addition to the favourable biomechanical properties of the AI technique, biological augmentation may also contribute to improved healing rates [5, 14, 24, 25].

Improved postoperative clinical outcomes have been reported in the literature following both BHT and RT repairs, and our findings are consistent with this evidence [4, 6, 7]. However, in the current study, a lower proportion of patients with chronic tears (>3 months) achieved the IKDC PASS [15] compared with those treated in the acute setting (54.1% vs. 91.7%; *p* = 0.0094). Moreover, patients with chronic lesions reported higher VAS scores than those with acute lesions (3.0 ± 1.8 vs. 1.6 ± 2.1; *p* = 0.0169). These findings are consistent with previous reports indicating that a shorter interval between injury and surgery is associated with improved PROMs [20].

Notably, the current study found a higher rate of RTSs (87.2%) after AI repair. This finding is clinically relevant, as it may reflect the translation of the biomechanical advantages previously described for the AI technique into meaningful clinical outcomes.

Another noteworthy finding was that, in addition to medial‐sided injuries, isolated meniscal repair was identified as a poor prognostic factor compared with repairs performed concomitantly with ACL reconstruction. There are several important concepts to highlight when discussing the differences between isolated meniscal repair and meniscal repair performed concomitantly with ACL reconstruction. First, a growing body of evidence suggests that the biological environment created during ACL reconstruction may enhance meniscal healing. Drilling of the tibial and femoral tunnels during ACL reconstruction introduces marrow elements, including growth factors and pluripotent cells, into the joint, which may positively influence the reparative response at the meniscal repair site, potentially contributing to improved healing outcomes in combined procedures compared with isolated repairs. Studies have reported higher healing rates and more favourable MRI signal characteristics following meniscal repair with concomitant ACL reconstruction, consistent with this biological hypothesis [3].

Second, ACL reconstruction improves biomechanical stability of the knee, reducing excessive translational and rotational shear stresses that can adversely affect meniscal repair integrity. Enhanced joint stability may lessen mechanical strain across the repaired meniscus, which can facilitate more robust tissue integration and lower failure rates in combined procedures. Clinical series in the sports orthopaedic literature have consistently shown lower failure and reoperation rates when meniscal repair is performed at the time of ACL reconstruction compared with isolated repair, supporting this biomechanical advantage [19, 20]. However, the literature on the influence of concurrent ACL reconstruction on clinical outcomes remains inconclusive, and emerging studies continue to shed light on the potential clinical benefits of concomitant ACL reconstruction [1, 16, 29].

Conversely, a systematic review specifically investigating predictors of repair failure found that studies of higher methodological quality were more likely to support the protective effect of concomitant ACL reconstruction [31]. One proposed mechanism underlying this protective effect is the biological augmentation provided by growth factors released during ACL reconstruction, which may enhance meniscal healing [5]. Additionally, patients undergoing concomitant ACL reconstruction typically follow a more cautious and protective rehabilitation protocol, which may further support healing of the repaired meniscus.

This study has several limitations. First, its retrospective comparative design allows for hypothesis generation but does not permit causal inference. Second, the data were obtained from a single centre and a single surgeon, which may limit the generalisability of the findings. Third, only lateral‐sided injuries were included in the RT group, which may introduce selection bias. Moreover, the heterogeneity of the cohort made a separate analysis of PROMs and failure rates between males and females irrelevant, as unequal subgroup sizes would not have allowed for statistically significant results. Moreover, since the five patients that experiences a failure were not included in the PROMs assessment, this could have introduced a selection bias, leading to possibly more optimistic results. Fourth, a formal sample size calculation was not performed due to the retrospective design. Although a larger sample would increase statistical power for subgroup comparisons, our cohort size is consistent with other published studies available in the literature. Finally, PROMs and satisfaction were assessed only among cases without failed meniscal repair, which may have introduced bias, particularly in the evaluation of patient satisfaction.

Further studies with larger cohorts and balanced cohorts will be useful to assess sex‐related differences in failure rates in these kind of meniscal lesions.

Despite these limitations, the present study holds clinical relevance, as it provides new evidence on the outcomes of complex meniscal tear repairs using a specific surgical technique.

## CONCLUSIONS

Meniscal repair of BHTs and RTs achieved 98% patient satisfaction and high return‐to‐sport rates with a 17% mid‐term failure. Medial BHTs and degenerative lesions demonstrated a higher risk of failure, whereas repairs performed concomitantly with ACL reconstruction showed lower failure rates. These findings support early repair and highlight the importance of tear pattern, aetiology, lesion chronicity and concomitant procedures in predicting outcomes.

## AUTHOR CONTRIBUTIONS

Maria Pia Neri, Maria Pia Neri and Stefano Zaffagnini had the initial idea of the study and supervised the work by reviewing the paper and conducting the decision‐making processes. Federico Maria Adravanti, Claudio Rossi and Luca Ambrosini contributed to Material preparation, data collection and analysis. Luca Ambrosini and Ashraf Hantouly contributed to the article by writing the first draft of the manuscript.

## CONFLICT OF INTEREST STATEMENT

Prof. Stefano Zaffagnini reports a relationship with Smith & Nephew Inc and Johnson & Johnson MedTech that includes consulting or advisory and is the editor‐in‐chief of the Journal of Experimental Orthopedics (JEO). Alberto Grassi reports a relationship with Smith & Nephew Inc and Johnson & Johnson MedTech that includes consulting or advisory. The remaining authors declare that they have no known competing financial interests or personal relationships that could have influenced the work.

## ETHICS STATEMENT

Approval was obtained from the ethics committee of AVEC (Area Vasta Emilia Centro) with approval number 645/2025/Oss/IOR. The procedures used in this study adhere to the tenets of the Declaration of Helsinki. Informed consent was obtained from all individual participants and parents of participants in case of minors included in the study.

## Supporting information

STROBE‐checklist‐Complex meniscal lesions.

## Data Availability

The data that support the findings of this study are available from the corresponding author upon reasonable request.
